# Immediate Esthetic Rehabilitation of Periodontally Compromised Anterior Tooth Using Natural Tooth as Pontic

**DOI:** 10.1155/2016/8130352

**Published:** 2016-04-19

**Authors:** K. Pavan Kumar, Surya Kumari Nujella, S. Sujatha Gopal, K. Karthik Roy

**Affiliations:** Conservative Dentistry & Endodontics, MNR Dental College and Hospital, Sangareddy, Telangana 522094, India

## Abstract

For patients who require removal of anterior teeth and their replacement various treatment modalities are available. With advancement in technology and availability of glass/polyethylene fibres, use of natural tooth as pontic with fibre reinforced composite restorations offers the promising results. The present case report describes management of periodontally compromised mandibular anterior tooth using natural tooth pontic with fibre reinforcement. A 1-year follow-up showed that the bridge was intact with good esthetics and no problem was reported.

## 1. Introduction

Dentists occasionally face complex situations that warrant removal of teeth from high esthetic zone. Despite wide range of treatment options that can be provided to conserve the tooth, extraction of an single anterior tooth is inevitable in case of trauma, advanced periodontal disease, root resorption, or failed endodontic therapy [1]. Following loss of the anterior tooth, it is important that an immediate replacement is provided in order to avoid esthetic, masticatory, and phonetic difficulties and to maintain the edentulous space [2]. Also the missing anterior tooth will have serious psychological implications on the patients. The treatment options for such situations should include the immediate replacement of the missing tooth which has to fulfil the cosmetic demands and functional needs and should be conservative, minimally invasive, biocompatible with permitting of oral hygiene maintenance.

Conventionally, the solution to this clinical problem has been the provision of a single tooth, removable temporary acrylic prosthesis, implants, or resin bonded bridges [3, 4]. Each method has its own advantages, disadvantages with varying levels of patient acceptance. Using the natural tooth as a pontic in this scenario has advantages of being the right size, shape, and color with a high level of patient acceptance due to the positive psychological value of using his or her natural tooth [5]. However use of natural tooth as pontic is not new technique; it was described 35 years earlier [6]. Despite the advantages of this clinical approach, it does not seem to be widespread in daily dental practice due to the compromised results over long a period of time. With the utilisation of newly advent fibre reinforcement technique use of natural tooth as pontic provides promising results. The present case report describes using natural tooth as pontic with fibre reinforcement.

## 2. Case Report

**Figure 1:**
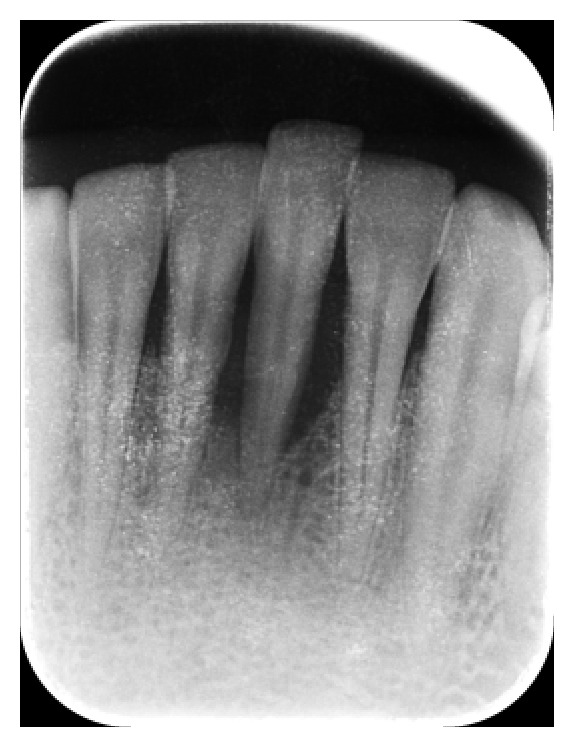
Preoperative radiograph.

A 30-year-old healthy male patient reported to the Department of Conservative Dentistry and Endodontics, MNR Dental College and Hospital, with a chief complaint of the mobility of mandibular leftcentral incisor. Upon clinical examination tooth was grade III mobile one with probing depth of 8 mm. Diagnostic radiograph of the tooth revealed bone loss around the mandibular left central incisor (Figure 1). The diagnosis was chronic localized periodontal disease. All the possible treatment modalities were explained to the patient. As the patient was highly concerned with esthetics, the possibility of using the clinical crown as a natural pontic was proposed.

**Figure 2:**
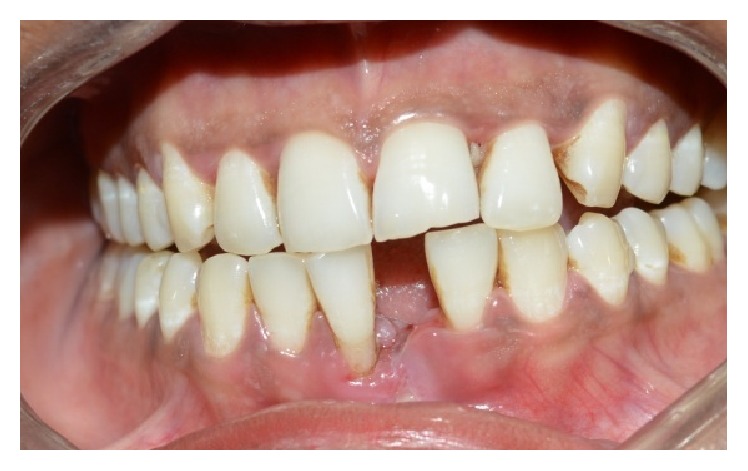
Atraumatic extraction.

**Figure 3:**
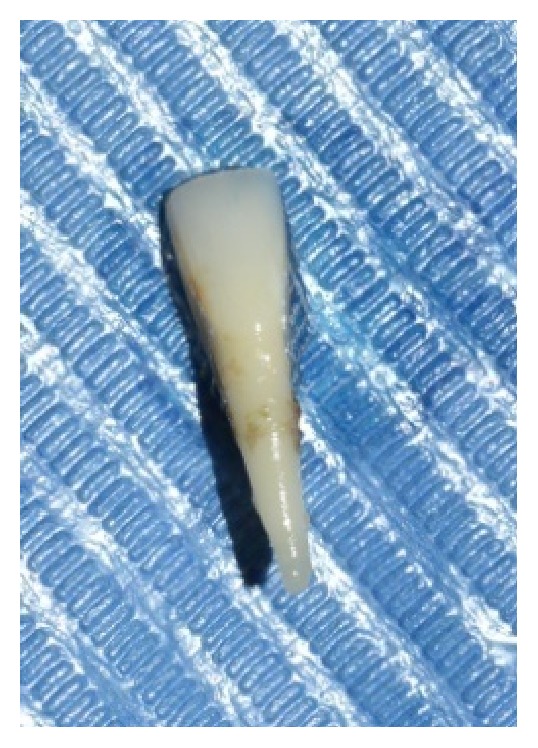
Extracted tooth.

**Figure 4:**
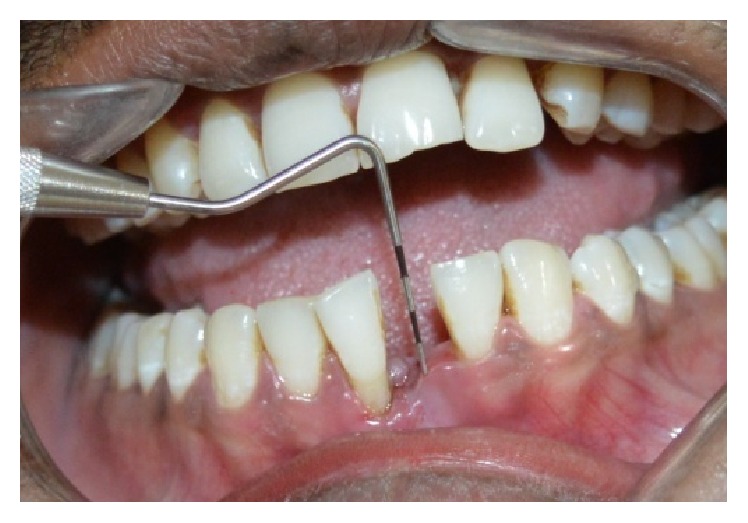
Space measurement for pontic.

**Figure 5:**
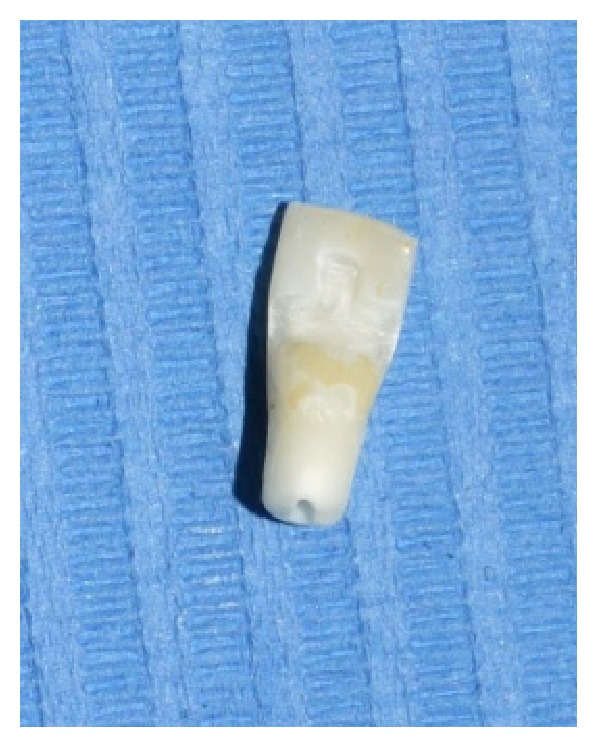
Pontic preparation.

The tooth was extracted under local anaesthesia atraumatically (Figures 2 and 3) and haemostasis was achieved. The length of the natural tooth pontic was determined by measuring the distance from incisal edge of central incisor to the extraction site with periodontal probe (Figure 4). An additional 2 mm length was added to the pontic to compensate for the gingival recession during healing phase. The tooth was sectioned (Figure 5) to the measured length and root canal was instrumented and debrided retrogradely. Copious irrigation with 5.25% of sodium hypochlorite was done to dissolve any pulpal remnants, if present. The canal was dried with the paper points and then sealed with flowable composite resin (G-ænial flo GC America). A modified ridge lap shape was given to the cervical area of the pontic as it provides both oral hygiene and esthetics [5].

**Figure 6:**
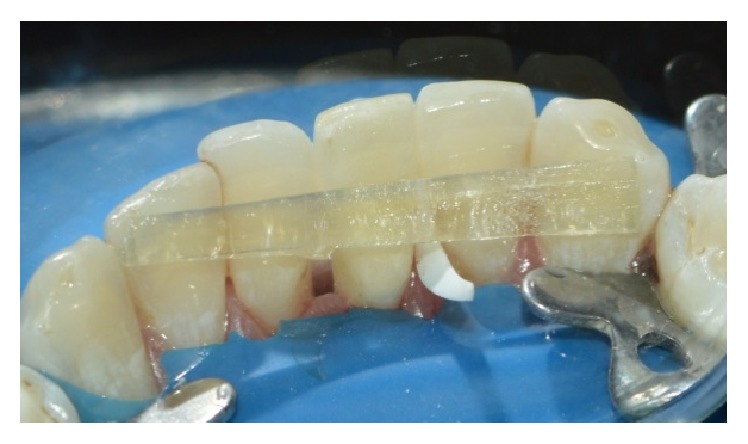
Trial placement of fibre.

**Figure 7:**
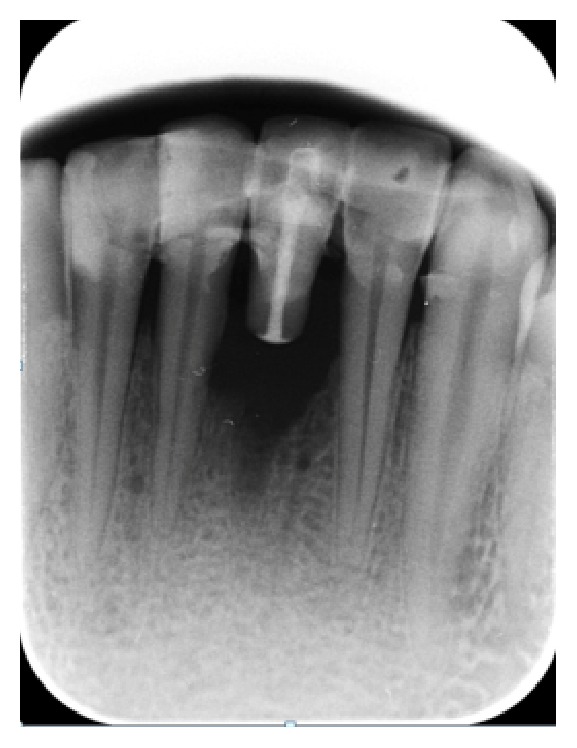
Postoperative radiograph.

**Figure 8:**
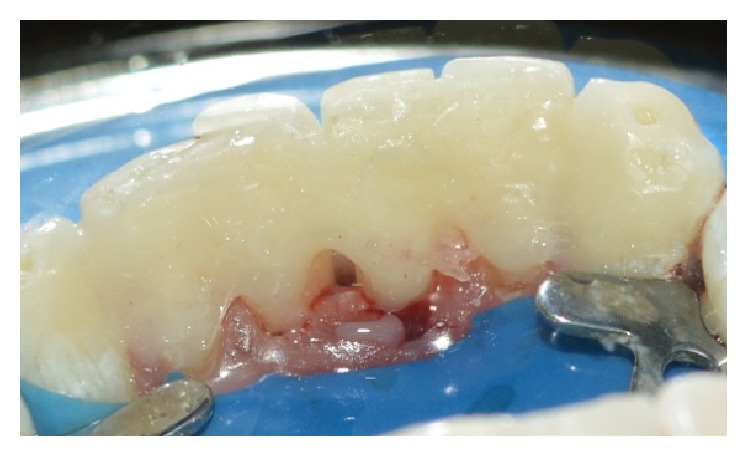
Postoperative palatal view.

**Figure 9:**
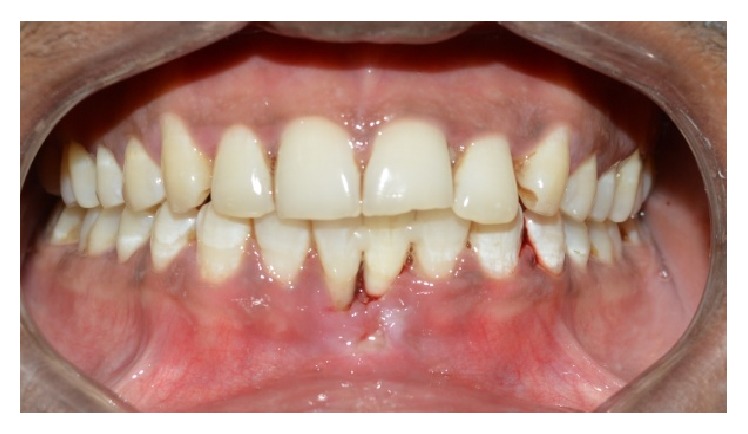
Immediate postoperative photograph.

**Figure 10:**
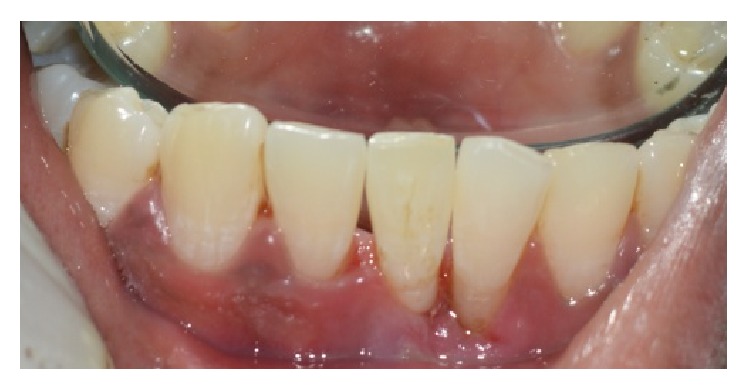
Recall visit, 1 year.

The required length of fibre strip (everStick crown and bridge material, Stick Tech Ltd., Finland) was measured and cut. Recess grooves were prepared on the lingual surface of pontic and adjacent abutment teeth were roughened. The natural tooth pontic was stabilized with flowable composite resin (G-ænial Universal flo GC America) on the facial surface and the wedge was placed to preserve the interdental space. Prepared surface was acid etched; bonding agent was applied and cured. A thin layer of flowable composite (G-ænial Universal flo GC America) was placed across the grooves and abutment teeth. Precut fibre was then placed in position and pressed into the prepared grooves for its close adaptation and cured (Figure 6). A further layer of composite was dispensed on the surface of fibre and cured, ensuring that all of the fibre surface was covered by composite and not exposed directly to the oral cavity. Excess composite resin was removed and occlusal interferences were again checked in protrusion and lateral excursions. Finishing and polishing were done to remove any rough surface, if present which might result in plaque retention. Immediate postoperative radiograph (Figure 7) and photographs were taken (Figures 8 and 9). Patient was recalled after every 3 months for the postoperative evaluation. A 1-year evaluation showed that the bridge was intact with good esthetics and no problem was reported (Figure 10).

## 3. Discussion

Various treatment options were available for the replacement of edentulous space created as a result of removal of single tooth in anterior esthetic zone. Each option has its own advantages and disadvantages. Acrylic removable partial dentures which can be placed immediately after the tooth are extracted. But they may be bulky, unesthetic, uncomfortable for the patient and inadequately preserve the extraction socket which may impede healing result in loss of soft and hard tissues [7]. The Fixed Partial Dentures (FPD) are widely proposed treatment option in such case due to their high strength and esthetic appearance compared to removal dentures. But they require aggressive tooth preparation of adjacent tooth for abudment, which may pose a high risk for pulp exposure especially in lower anterior teeth. Implant supported prostheses are more conservative in nature and first line of treatment. But they are more invasive, traumatic, and expensive. Moreover in this particular scenario compromised periodontal condition may compromise the long term prognosis and also does not match patients financial condition.

The use of natural tooth as a pontic was described in literature long back. However in those earlier days these pontics were connected to the adjacent teeth with adhesive composite resins, wire, metal mesh, nylon mesh, and cast metal frameworks bonded to the adjacent teeth. The inherent problems with these materials were their inability to be chemically incorporated into the composite resin and they could not support the repeated loading stresses placed on the bridge during normal and parafunction. When the thickness of composite resin used for bonding was increased it resulted in an increase in food and plaque retention subsequently poor oral hygiene [8]. Also due to low fracture strength of the bonded composite resin, if pontic debonds unexpectedly will results in an unpleasant social situation.

This challenge to place a thin but strong, bondable composite resin bridge was met with introduction of fibre reinforcement by creating a chemical bond between the strengthening fibre and composite [9, 10]. These materials have similar elasticity to dentin, thus distributing the mechanical stress concentrated within the connector to a wider area and diminishing the risk of failure.

EverStick crown and bridge fibre (Stick Tech Ltd., Finland) consist of individual silane glass fibres. The fibres are locked to each other with linear polymers (PMMA) and cross-linking monomers (bis-GMA) to form unique and patented Interpenetrating Polymer Network or IPN structure. IPN ensures both micromechanical and chemical bonding of everStick fibres to composites, adhesives, or composite cements. The bond strength is based on the ability of the polymer matrix to partially dissolve in the resin used for bonding. The significance of this is that surfaces can be reactivated even after final polymerisation. This reactivation dissolves linear polymers and forms new chemical bonds. The resin can also penetrate deeper into the fibre matrix which improves the micromechanical retention. With these crown and bridge fibres it was possible to produce a thin but strong, composite resin bonded bridge in a single visit. This prosthesis has been referred to as Resin Bonded Fibre Reinforced Composite Fixed Partial Denture or FRC FPD. These bridges show rigidity and flexural strength 7 times that of composite resin alone [11].

## 4. Conclusion

The use of fibre composite to bond natural tooth to adjacent teeth in case of isolated single tooth loss appears to be promising treatment option in immediate esthetic rehabilitation. The advantages of natural tooth pontic are its excellent shade matching, life-like translucency, and patients superior psychological acceptance.
